# Molecular Motifs in Vascular Morphogenesis: Vascular Endothelial Growth Factor A (VEGFA) as the Leading Promoter of Angiogenesis

**DOI:** 10.3390/ijms241512169

**Published:** 2023-07-29

**Authors:** Claudiu N. Lungu, Mihaela C. Mehedinti

**Affiliations:** Departament of Functional and Morphological Science, Faculty of Medicine and Pharamacy, Dunarea de Jos University, 800010 Galati, Romania

**Keywords:** vascular morphogenesis, VEGFR, VEGF-A, angiogenesis, protein docking, conformational analysis, molecular descriptors

## Abstract

Tissular hypoxia stimulates vascular morphogenesis. Vascular morphogenesis shapes the cell and, consecutively, tissue growth. The development of new blood vessels is intermediated substantially through the tyrosine kinase pathway. There are several types of receptors inferred to be located in the blood vessel structures. Vascular endothelial growth factor A (VEGF-A) is the leading protagonist of angiogenesis. VEGF-A’s interactions with its receptors VEGFR1, VEGFR2, and VEGFR3, together with disintegrin and metalloproteinase with thrombospondin motifs 1 (ADAMTS1), connective tissue growth factor (CTGF), and neuropilin-1 (NRP1), independently, are studied computationally. Peripheral artery disease (PAD), which results in tissue ischemia, is more prevalent in the senior population. Presently, medical curatives used to treat cases of PAD—antiplatelet and antithrombotic agents, statins, antihypertensive remedies with ACE (angiotensin-converting enzyme) impediments, angiotensin receptor blockers (ARB) or β- blockers, blood glucose control, and smoking cessation—are not effective. These curatives were largely established from the treatment of complaint cases of coronary disease. However, these medical curatives do not ameliorate lower limb perfusion in cases of PAD. Likewise, surgical or endovascular procedures may be ineffective in relieving symptoms. Eventually, after successful large vessel revascularization, the residual microvascular circulation may well limit the effectiveness of curatives in cases of PAD. It would thus feel rational to attempt to ameliorate perfusion in PAD by enhancing vascular rejuvenescence and function. Likewise, stimulating specific angiogenesis in these cases (PAD) can ameliorate the patient’s symptomatology. Also, the quality of life of PAD patients can be improved by developing new vasodilative and angiogenetic molecules that stimulate the tyrosine kinase pathway. In this respect, the VEGFA angiogenetic pathway was explored computationally. Docking methodologies, molecular dynamics, and computational molecular design methodologies were used. VEGFA’s interaction with its target was primarily studied. Common motifs in the vascular morphogenesis pathway are suggested using conformational energy and Riemann spaces. The results show that interaction with VEGFR2 and ADAMTS1 is pivotal in the angiogenetic process. Also, the informational content of two VEGFA complexes, VEGFR2 and ADAMTS1, is crucial in the angiogenesis process.

## 1. Introduction

The tissue environment plays a critical role in modeling the angiogenesis process. Integrins regulate these interactions [1,2]. Vessel formation is necessary to help vascular networks develop and thus help embryogenesis. The dothelial and perivascular cells are crucial in vessel development [3,4]. Fibronectin (FN), an extracellular matrix protein, is involved in the vascular morphogenesis of perivascular cells. Furthermore, fibronectin is involved in cardiovascular development. Lethal vascular malformations are noted in cases of FN absence [5,6]. In FN-null embryos derived from the 129S4 strain of mice, endothelial cells are present within the embryo. However, they are not assembled into dorsal aortae, indicating the necessity for FN in vasculogenesis [7,8,9]. In this respect, a null mutation within the FN1 gene on the zebrafish mutant proves that FN1 is crucial to forming one heart tube from the bilateral cardiac primordia [10]. Additionally, FN is critical for cell polarity within cardiac precursors [11]. These molecules are closely related in their function to fibrin. The αv subunit of integrin binds FN and plays distinct roles during embryonic development. Endothelial cells in which all β1-containing integrins are deleted showed that these cells still bind to FN, while they are unable to bind collagen or laminin, substrates for α1β1 and α2β1 integrins [12,13]. Immunohistochemical analyses proved that FN proteins are dominant in the dorsal aortae and parts of arteries within the anterior trunk of embryos [14]. Additionally, dorsal aortae are the primary embryonic vessels to be invested (containing) with alpha-smooth muscle actin (αSMA)-expressing vascular smooth muscle cells (VSMCs) [15]. Their role is demonstrated in the following processes: (1) the migration of VSMC pro-genitors to the dorsal aorta; (2) differentiation of perivascular cells to obtain aortic VSMC characteristics; and (3) proliferation and survival of VSMCs or their precursors. FN and its receptors are essential. The absence of all FN forms leads to a defective association of aortic endothelial cells with the adjacent mesenchyme [16]. If mRNAs for FN and its splice variants are produced by the mesenchymal cells adjacent to the dorsal aortae, it would be expected that the receptors for FN are expressed on the endothelial cells. Indeed, during vascular remodeling following balloon catheterization, FN and its splice variants are expressed by the proliferating VSMCs and receptors known or believed to bind FN’s arginyl glycyl compound (RGD) [17,18]. The involvement of endothelial and perivascular cells is crucial during embryogenesis and after birth for physiological and pathological processes requiring new vessel formation or remodeling [19,20]. Similarly, endothelial and perivascular cells are associated with vessel stability [21]. Several general signaling pathways control endothelial cell (EC) behavior during angiogenic sprouting, including TIE2 and Notch signaling—as does signaling via known axon guidance receptors expressed in ECs. However, the secreted vascular endothelial protein A (VEGFA), also called vascular permeability factor (VPF), is the master regulator of recent vessel sprouting during development, growth, and disease. VEGFA is the best-characterized member of a family of homodimeric glycoproteins, including placental protein (PLGF), VEGFB, VEGFC, and VEGFD [22]. During angiogenesis, VEGFA binds to its cognate receptor Tyr kinase and VEGF receptor-2 (VEGFR2), also called KDR and FLK1, and activates multiple downstream pathways via signaling intermediates, like mitogen-activated protein kinases (MAPKs), phosphoinositide 3-kinases (PI3Ks), AKT, phospholipase Cγ, and little GTPases [23]. 

Furthermore, VEGF initiates EC proliferation, degradation of the extracellular matrix, and, more importantly, the chemotaxis process [24]. Hypoxia stimulates VEGFA expression, so a rapid angiogenetic response is conducted in the case of tissular hypoxia [25]. Signaling via the VEGFR is pro-angiogenetic to some extent. Migration is induced by the interaction of the TIE2 receptor with matrix-associated angiopoietin 1 (ANG1) [26]. Also, alternative pathways like Tyr phosphatase (VE-PTP, also called PTPRB) are to be noted [27,28].

Remodeling of the vessel wall is also implied in angiogenesis. ANG2 antagonizes ANG1 activity on TIE2 to destabilize vessels and aid angiogenic remodeling. Homophilic VE–cadherin interactions maintain EC–EC junctions. Delta-like 4 (DLL4)-mediated activation of Notch receptors represses angiogenic cell behavior and promotes vessel stability upon the proteolytic release of the Notch intracellular domain (NICD). Roundabout homolog 4 (ROBO4) mediates the inhibition of VEGFR signaling and thus acts like a process signaling pathway to maintain vessel integrity [29,30].

Furthermore, EPHB4–ephrin B2 signaling is involved in vascular development. This complex allows for VEGFR2 or VEGFR3 membrane internalization and amplifies angiogenetic signaling) [31,32,33]. Cell hierarchical organization also plays a role in angiogenesis. Sprouting endothelial cells are hierarchically organized into leading tip cells (TCs) and trailing stalk cells (SCs) that exhibit distinct and specialized cell behavior [34,35]. 

The most studied and essential pathway is the VEGFR2 pathway. VEGFR2 activation induces DLL4 expression in TCs, which activates Notch on adjacent SCs and VEGF-mediated disruption of a repressive translocation ETS leukemia (TEL) and carboxy-terminal-binding protein (CtBP) complex [36]. Also, recent findings have expanded data regarding receptor tyrosine kinase (RTK) regulation. Mainly, G-protein-coupled receptors play a crucial role in the angiogenetic pathway. Inhibiting RTKs will be a promising therapeutic strategy for vascular disorders [37].

Regarding VEGFR3, Notch signaling in SCs downregulates its expression. Notch-induced Notch-regulated ankyrin repeat-containing protein (NRARP) expression enhances WNT signaling in SCs, maintaining EC–EC junctions, promoting proliferation, and augmenting DLL4 expression via β-catenin [38]. 

Also, feedback inhibition of Notch signaling is performed by NRARP. All VEGF members bind to tyrosine kinase receptors on the cell surface, producing a dimerization, and are thought to become active through transphosphorylation [39]. 

There are three sorts of VEGFR: VEGFR 1-3. VEGFR1 interacts mainly with VEGFA. VEGFR2 mediates the cellular responses to VEGF, and VEGFR1 modulates VEGFR2 signaling. VEGFR1 also acts as a dummy receptor for VEGF. VEGFR-3 regulates lymphangiogenesis in response to VEGF-C and VEGF-D. As stated above, the angiogenesis pathway supported by VEGFA can be complex. Multiple interactions are required to initiate and shape the vascular morphogenesis process. The VEGFR system supports the leading process dynamic. Interacting with all VEGFR members (with the identical mode of action (MOA)) finally results in distinct effects on vascular morphogenesis. Some essential features offer each VEGFR type distinct properties [40]. 

This computational research studies VEGFA’s interaction with its designated receptors and some related molecules that bind VEGFa. Computational methods were used to quantify and characterize the interaction between VEGFA and its targets. VEGFA’s interactions were studied in light of the information content and chemical spaces using molecular descriptors and Riemann surfaces.

## 2. Results

**Figure 1:**
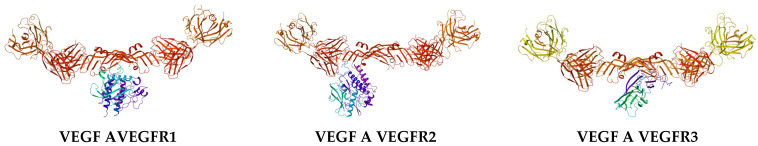
VEGFA in complex with VEGFR1, 2, and 3. The most favorable energetic conformations are represented. The molecules are represented as the ribbons’ technical view. It was observed that VEGFA binds to VEGFR-type receptors in distinct regions. (VEGFA is significantly heavier and bigger than VEGFR1,2,3). In all poses, water molecules had been removed.

Peptide–peptide docking retrieved more than 100 stable conformations for each of the complexes. As a result, 96 protein–protein complexes were selected for each receptor. In Figure 1, the best poses for each complex are shown in the ribbons’ technical view. 

**Figure 2:**
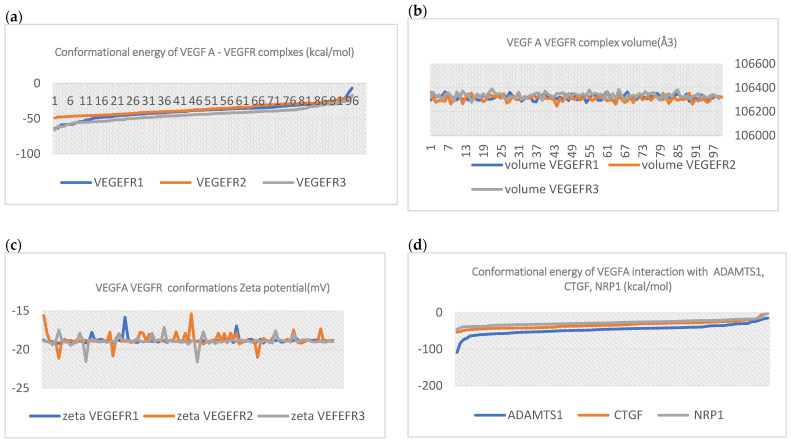
(**a**) VEGFA-VEGFR1,2,3, complex energies are shown. The energy for each receptor is represented for every 96 VEGFA-VEGFR conformations. For example, the VEGEFR2 receptor in complex with its ligand (VEGFA) has medium conformational energy compared to VEGFR1 and 3; (**b**) volume (Å3) of VEGFA-VEGFR complexes. VEGFA in complexes with VEGEFR 2 and 3 has wider variation, whereas the complex VEGFA-VEGFR 1 seems to explore a relatively stable domain; (**c**) zeta potential of VEGFA-VEGEFR conformations; (**d**) the conformational energy of VEGFA during interaction with it is molecular targets: ADAMTS1, CTGF, and NRP1 (kcal/mol). ADAMTS1 has the lowest interaction energy counted for 96 molecular conformations.

In Figure 2, VEGFA–VEGEFR1,2,3, complex energies are shown. The energy for each receptor is represented for every 96 VEGFA-VEGEFR conformations. For example, the VEGEFR2 receptor in complex with its ligand (VEGFA) has medium conformational energy compared to VEGEFR1 and 3. 

Regarding the volume of the VEGFA–VEGEFRB complexes’ conformational states, each complex volume is represented in Figure 2. The volume of all complexes has slight variation through all of the conformations. Complex solubility, the zeta potential variation, is also represented. All of the complex zeta values suggest rapid coagulation or flocculation. 

**Figure 3:**
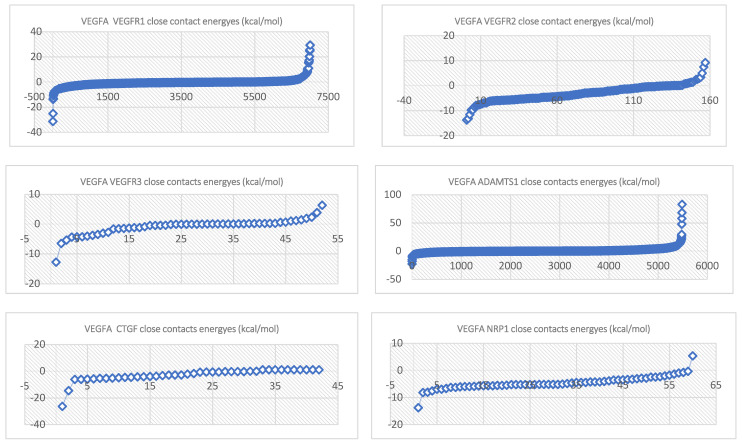
Close-contact energies (kcal/mol) of VEGFA during interaction with VEGEFR1,2,3 and ADAMTS1, CTGF, and VEGFA (the VEGFA-VEGEFR1 and VEGFA-ADAMTS1 complexes have the most energetically favorable and most significantly energetically favorable populations, respectively). VEGEFR3, CTGF, and NRP1 have inferior close-contact populations.

ADMTS-1, CTGF, and NRP1, along with VEGEFR complex energies, are shown in Figure 3.

The close-contact energy trendline equations are represented in Table 1. 

In Table 2, the Riemann surfaces generated from the polynomial equations (Figure 3) are represented together with the 2D Riemann surface schematization.

Atom information content (mean) is the entropy of element distribution in the molecule, where *n_i_* is the number of atomic number (i) occurrences in the molecule. Also, *p_i_* = *n_i_*/*n*, where *n* is the sum of the *n_i_*. The value of a_ICM is the negative of the sum over all *i* of *p_i_* log *p_i_*. Atom total information content is computed to be a_ICM times *n*. Vertex adjacency information (number of magnitudes): 1 + log_2_ *m*, where *m* is the number of heavy–heavy bonds. Vertex adjacency information is computed using the formula: −(1 − *f*)log_2_(1 − *f*) − *f* log_2_ *f*, where *f* = (*n*^2^ − *m*)/*n*^2^, *n* is the number of heavy atoms and *m* is the number of heavy–heavy bonds(Table 3) [41,42,43,44].

The total information content and mean information content of each VEGFA complex represented in Table 3 show equal values for the VEGFA complex with VEGEFR2 and ADAMTS1. The computed equations show that both complexes operate with the same biological information and presumably the same biological activity. The vertex adjacency information-based index retrieved the same result for all conformational poses of each complex. 

The entropy obtained for each complex is as follows: VEGFA–VEGEFR1; VEGFA–VEGEFR2 = 25.0591; VEGFA–VEGEFR3 = 42.638 (for all complex conformations); VEGFA–ADAMTS1; VEGFA–CTGF; VEGFA–NRP1.

Considering the interaction dimensionality computed with Riemann spaces using the close-contact energy, all VEGFA complexes have the same vertex adjacent information, meaning that they have the same dimensionality but are distinct in their information complexity judging by the information indices. 

**Figure 4:**
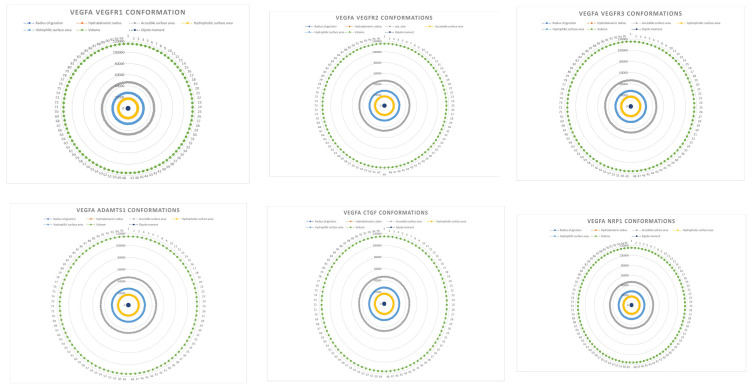
VEGFA complexes’ properties. The radius of gyration, accessible surface area, hydrophobic surface area, hydrophilic surface area, volume complex volume, and protein dipole moment are graphically represented as radar plots.

Regarding protein structural information, some properties computed like the area of hydrophobic protein residues, the area of positive protein residues, the area of negative protein residues, the area of ionic residues, net charges, and protein mass (ligan–receptor complex mass) have no variation or slight variation through the complexes conformations. For example, the VEGFA–VEGFR1,2,3, ADAMTS1, CTGF, and NRP1 complex mass values are as follows: 116,9536; 116,9496; 116,956; 116,9476; 116,9557; and 116,9516 (kDa); furthermore, the other protein-related properties vary slightly in all other conformation populations. In Figure 4, some protein-related properties are represented as radar plots.

## 3. Discussion

The interaction between the two molecules (VEGFA and its receptors VEGEFR1,2,3, ADAMTS1, CTGF, and NRP1) finally ends up in a complex mathematical system shown by the equations in Table 3 with roots with complex solutions. The graphical representations of the equations are performed with the assistance of the Riemann sum using the subsequent formula: S = $\overset{n}{\underset{i=l}{\sum }}f\left(xi\right)\Delta xi,$ where Δxi = xi − 1 and xi∈[xi − 1, xi] and xi and xi − 1 are the ends of the Riemann function interval. Furthermore, the entire existence intervals of polynomial functions end within the Riemann function existence intervals. For the Cartesian system, the function representations are shown in Table 2. It was observed that the function values are between the (+∞,−∞) limits on the oz axis. The fixed points of the graph are the equation roots. Applying the Riemann sum on the subinterval of the definition of the polynomial function leads to the complete function graph. While the function roots are real and sophisticated, the function graph covers much of the function definition space. Each subinterval includes a maximum of the polynomial functions. Therefore, the full-function graph shows a satisfying aspect of hyperboloids. By analyzing the contact energies, it was observed that the contact molecule position is significant. Overall, the binding position from the two molecules that form the complex finishes up in energy the larger the binding site is near the ox axis. The function form is mathematically given by the function graph concerning the function definitions domain in real and complicated (imaginary) space.

Simple graphs are digraphs with undirected edges. For example, two vertices in an exceedingly simple graph are considered adjacent if they are the endpoints of the very edge. Entropy quantifies the quantity of missing information before the reception. By Shannon, entropy is expressed in terms of a discrete set of probabilities (pi): (X) = −$\overset{n}{\underset{i=1}{\sum }}p\left(xi\right)logp\left(xi\right)$. Information entropy and thermodynamic entropy are directly correlated [45,46,47,48,49,50]. Both expressions describe the same phenomena. Entropy is characterized by the Boltzmann relation: H = klog(W) (K units/nat), where W is the number of microstates and H is the entropy. The Boltzmann constant K may be interpreted as the thermodynamic entropy per nat [51] Also, the entropy expressed in nats is H = −$\overset{W}{\underset{i=1}{\sum }}plog\left(p\right)=log\left(W\right)$. 

As stated in the introductory part, the thyroxin kinase pathway is involved in all members of VEGF molecules. Their mode of action is similar in all families of molecules—forming bonds by dimerization. VEGF receptors possess an extracellular domain and an intracellular domain with a tyrosin-kinase-specific site. Regarding each VEGFR function, the function of VEGFR-1 is not entirely defined, although it is thought to modulate VEGFR-2 signaling [40,52,53,54]. Also, VEGFR-1 acts as a decoy for VEGF [55]. VEGFR2 is the primary molecule that interacts with VEGF and promotes angiogenesis. 

VEGFR-3 mediates lymphangiogenesis in correlation with VEGF-C and VEGF-D [51,52]. Also, other receptors interact with VEGF. Neuropilins (NRP) are pleiotropic receptors that interact with VEGF [40,53]. Regarding ADAMTS1, it has been shown that two ADAMTS proteins proven to be antiangiogenic are ADAMTS-1 and ADAMTS-8. Both can inhibit VEGF-induced angiogenesis during a chick tissue assay and suppress fibroblast-growth-factor-2 [56,57,58,59,60,61].

Also relevant to the antiangiogenic actions of ADAMTS-1 is that TSP1 can interact with CD36, a membrane glycoprotein receptor on endothelial cells [62,63]. This involves the sequence CSVTCG in two of the three TSs [64]. Another motif is the GWQRRL/TVECRD motif common to the primary C-terminal TS repeat of both ADAMTS-1 and -8 but absent from the alternative ADAMTS, which may play a significant role. 

Furthermore, the aggrecanases ADAMTS-1, -4, -5, -8, -9, and -15 aggrecan is the foremost important proteoglycan responsible for flexibility [65]. It contains two N-terminal globular domains, G1 and G2, separated by an IGD (interglobular domain), followed by a GAG-attachment region and a C-terminal globular domain [66]. 

The G1 domain interacts with mucopolysaccharides and links proteins to make large aggregates trapped within the cartilage collagen matrix. Aggrecan protects cartilage collagen from degradation [67]. 

Regarding their active site, two major proteolytic cleavage sites are identified in this domain: one at Asn341–Phe342 and one at Glu373–Ala374. Regarding ADAMTS1’s absence, it was shown that its absence in Adamts1-null mice causes growth retardation, tissue malformation, and decreased fertility with changes within the histology of the uterus and ovaries [68,69]. Also, ADAMTS1 is a transcriptional target of the progesterone receptor during ovulation [70], and it is upregulated in bone and osteoblasts by secretion and related agents [71]. ADAMTS1 is downregulated in endothelial cells derived from the livers of cirrhotic animals [71] and is additionally reduced within the subcutaneous tissue of obese mice [72]. ADAMTS-4 may affect CNS pathology [73] and tumor pathology [74]. By stimulating ADAMTS1, angiogenesis is often promoted via the integrin and fibrin systems [75]. Furthermore, forming new blood vessels is a dynamic, highly regulated process that depends on coordinated signaling by growth factors and cell adhesion receptors [76]. 

Lastly, PAD’s prevalence exceeds 10 million people. PAD is typically caused by atherosclerotic obstructions within the large arteries of the leg(s). Also, despite extensive research, medical therapies to increase perfusion to the distal limbs are of limited benefit. Although early angiogenesis and cell therapy studies show some isolated results, the employment of angiogenesis and arteriogenesis therapies remains limited [77]. As shown by various studies, angiogenesis mediated by VEGF is crucial. Within the well-studied VEGF receptor–ligand family, VEGFR2 is the primary receptor whose activation drives hypoxia-dependent and post-natal angiogenesis [78]. Furthermore, VEGF-A may be a structurally related ligand for the VEGFR tyrosine kinases VEGFR1, R2, and R3 [78]. The computational study indicates that VEGFA–VEGFR and ADMT1’s interaction must support the development of new molecules. The interactions’ informational content, chemical space characteristics, and energy profile are often used further to develop specific angiogenesis and arteriogenesis therapy for PAD [79].

## 4. Materials and Methods

In order to explore the angiogenetic process, specifically VEGFA’s interaction with its known targets, the protein data bank (PDB) structures of VEGFA(1BJ1) [80] and its receptors VEGFR1(1FLT) [81], VEGFR 2(1Y6A) [82], and VEGFR3(4BSJ) [83], together with ADAMTS1(2JIH) [84], CTGF(Uniprot P29279) [85], and NRP1(1KEX) [86], respectively, were studied. The PDB structures were energetically minimized using the AMBER 99 force field [87]. The structures were protonated at pH = 7.4 and 310 K with a salt concentration of 0.9 NaCl (154 mmol/L Na^+^). In order to analyze VEGFA’s interaction with its targets, protein–protein docking studies were performed. VEGFA was considered as a ligand and VEGFR1,2,3 and ADAMTS1, CTGF, and NRP1 were considered as receptors. Docking evaluation was performed by considering 96 ligand–receptor complexes’ conformations for all six systems studied. The docking studies were implemented by using the Schrodinger software package [88]. For CTGF, a homology model was used based on uniport sequence P29279. The homology model was generated using the online SWISS PROT server [89]. After the model was developed, it was protonated and energetically minimized using the same protocol.

A conformational energy population was used to evaluate the interaction between VEGFA and its targets. By representing the conformational energy confrontations for all VEGFA complexes, a polynomial trendline was computed for each complex. In order to obtain 2D and 3D quantification, polynomial six-degree equations were used to generate Riemann surfaces. The 3D resultant Rieman surfaces were analyzed using their 2D diagrams. Finally, the Riemann surfaces and complexity maps were used to compare and assess the VEGFA protein interactions.

In order to validate the interactions, fingerprint protein–ligand interaction was used. Using fingerprint classification, a protein–ligand interaction fingerprint (PLIF) is a methodology for exploring the interactions between two proteins (one receptor and one ligand n. bond such as hydrogen, ionic interactions, and surface contact are organized after their Aa residue origin and built as fingerprints using a specific algorithm. The fingerprint data were generated using MOEa and Schrodinger software packages [90,91]. PLIF is widely used for characterizing protein–protein interactions, especially in molecular systems such as the G protein system [92]. Overall, PLIF gives dimensionality to an interaction, making assessments regarding binding affinities being used in docking procedures possible [89]. Conformation energies and contact energies were evaluated (kcal/mol). Contacts between VEGFA and the binding sites were analyzed using MOE software. Six types of contacts were identified: Hydrogen bonds, metal, ionic, arene, covalent, and Van der Waals distance interactions. Each contact was represented using scatter plots. A negative energy threshold was chosen to select the appropriate conformation. Conformation with energy higher than 3 kcal/mol was not considered [93].

Furthermore, the information content of the complexes was evaluated using informational molecular descriptors: total information content, mean information content, and vertex adjacency information (equal and mag). Also, a series of molecular descriptors were computed. 

Also, to characterize the molecular population studied, some protein properties were computed for each complex and conformation: the area of hydrophobic protein residues, the area of positive protein residues, the area of negative protein residues, the area of ionic residues, net charges, protein mass (complex mass), the radius of gyration, accessible surface area, hydrophobic surface area, hydrophilic surface area, protein volume (complex volume), and protein dipole moment. The properties were computed using an AMBER force field (at a PH target of 7.4, a temperature of 310,15 K, and a salt concentration of 0.9 NaCl). The computations were performed using the online server VCCL [94]. The radius of gyration, accessible surface area, hydrophobic surface area, hydrophilic surface area, protein volume (complex volume), and protein dipole moment were graphically represented, while those properties had slight preset variation following each complex conformation.

## 5. Conclusions

VEGF A interacts with multiple protein targets. The primary targets are its receptors VEGFR 1,2 and 3. Interactions with VEGFR are specific concerning each VEGFR type. For example, VEGFR1 interacts strongly with VEGFA, followed by VEFEGR types 2 and 3. Also, ADAMTS1 seems to have strong interaction with VEGFA. Considering the Riemann surfaces, VEGFR1 is the most complex interaction with VEGFA, followed by NRP1.

Regarding the information content of the VEGFA–receptor complexes, VEGFR2 and ADAMTS1, in interaction with VEGFA, carry virtually the same information. Finally, molecular motifs implied in angiogenesis share some common structural and energetical futures. Overall, by studying VEGFA’s interaction with its primary targets, crucial insights into molecular interactions will be obtained. In addition, studying this tyrosine-kinase-related pathway will endow the molecular design of a tyrosine-kinase-specific stimulator- that will promote angiogenesis and arteriogenesis. Furthermore, drawbacks like vascular mimicry (VM) stimulation by the novel compound must be avoided. Presumably, these novel therapies will improve PAD treatment and patients’ quality of life. 

## Figures and Tables

**Table 1:** Close-contact energy trendline equations of the most energetically favorable conformations.

Complex	Equation
VEGFA/VEGFR1	y = 21x^6^ − 16x^5^ − 13x^4^ − 10x^3^ −0.6x^2^ + 0.0104x − 8.2713
VEGFA/VEGFR2	y = −11x^6^ − 10x^5^ − 0.6x^4^ + 0.0003x^3^ − 0.0201x^2^ + 0.6414x − 12.746
VEGFA/VEGFR3	y = −0.8x^6^ + −0.6x^5^ − 0.0004x^4^ + 0.0138x^3^ − 0.2601x^2^ + 2.5731x − 12.569
VEGFA/ADAMTS1	y = −19x^6^ − 15x^5^ − 11x^4^ − 0.8x^3^ – 0.5x^2^ − 0.0186x
VEGFA/CTGF	y = −0.7x^6^ – 0.5x^5^ − 0.0052x^4^ + 0.1408x^3^ − 1.9297x^2^ + 12.493x − 34.148
VEGFA/NRP1	y = −0.9x^6^ − 0.7x^5^ − 0.5x^4^ + 0.0043x^3^ − 0.1242x^2^ + 1.5741x − 12.772

**Table 2:** Riemann surface (derived from the contact energy polynomial equation). 1. VEGFA–VEGEFR1 complex; 2. VEGFA–VEGEFR2 complex; 3. VEGFA–VEGEFR3 complex; 4. VEGFA–ADAMTS1 complex; 5. VEGFA–CTGF complex; 6. VEGFA–NRP1 complex. Left—Riemann surface schematics. Right—Riemann surface 3D representation.

Nr	Riemann Surface Schematics	Riemann Surface 3D
1	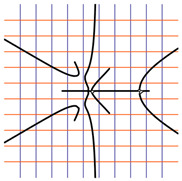	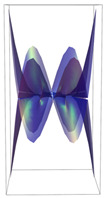
2	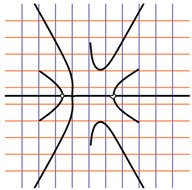	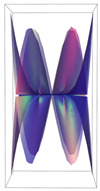
3	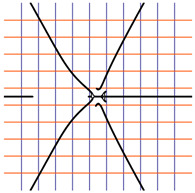	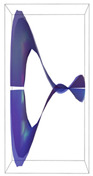
4	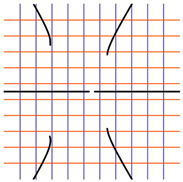	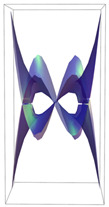
5	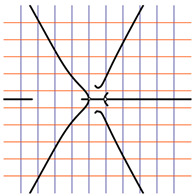	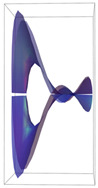
6	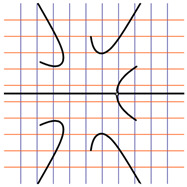	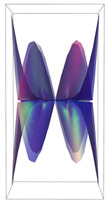

**Table 3:** Total information content, mean information content, and vertex adjacency information equal and magnitude.

Complex	Total Information Content	Mean Information Content	Vertex Adjacency Information Equal	Vertex Adjacency Information Mag
VEGFA–VEGFR1	30,187.6602	1.6931	0.0030	14.0463
VEGFA–VEGFR2	30,196.4746	1.6927	0.0030	14.0463
VEGFA–VEGFR3	30,190.5966	1.6930	0.0030	14.0463
VEGFA–ADAMTS1	30,196.4746	1.6927	0.0030	14.0463
VEGFA–CTGF	30,192.5586	1.6929	0.0030	14.0463
VEGFA–NRP1	30,184.7227	1.6932	0.0030	14.0463

## Data Availability

On reasonable demand.
